# A biophysical and molecular characterization of the interaction between the Alzheimer risk factor BIN1 and the neuronal scaffold protein p140Cap

**DOI:** 10.1016/j.jbc.2025.110665

**Published:** 2025-08-31

**Authors:** Danielle M. Blazier, Eric M. Lewandowski, Natasha Ram, Xiujun Zhang, Shuai Wang, Om Patel, Lisa Collier, Paola Defilippi, Yu Chen, Gopal Thinakaran

**Affiliations:** 1 Byrd Alzheimer’s Center and Research Institute, University of South Florida, Tampa, Florida, USA; 2 Department of Molecular Medicine, USF Morsani College of Medicine, Tampa, Florida, USA; 3 Department of Molecular Biotechnology and Health Sciences, University of Torino, Torino, Italy

**Keywords:** Alzheimer disease, BIN1, p140Cap, SRCIN1, Src homology 3 domain, protein–protein interaction, surface plasmon resonance, tau, rs138047593

## Abstract

Bridging integrator 1 (*BIN1*) is a genetic risk factor for late-onset Alzheimer disease. BIN1’s participation in endocytosis, membrane remodeling, and modulation of actin dynamics is well-characterized in non-neuronal cells. In neurons, BIN1 is enriched at presynaptic sites, where it facilitates excitatory neurotransmitter vesicle release. However, how BIN1 is involved in synaptic vesicle dynamics is not well understood. A C-terminal Src homology 3 (SH3) domain is invariant in all BIN1 isoforms and promotes protein–protein interactions with proteins harboring proline-rich motifs. While BIN1 interactions with dynamin, synaptojanin, RIN3, and tau have been identified and experimentally validated, the list of BIN1-interacting molecules is not exhaustive. Here, we report the neuronal scaffolding protein p140Cap, encoded by SRC kinase signaling inhibitor 1, as a BIN1 SH3 domain-interacting protein. We performed surface plasmon resonance to ascertain the affinity of BIN1-SH3 domain for p140Cap and identified a peptide containing three proline-rich motifs that exhibited biologically relevant affinity (K_D_ = 7.7 μM). Additional surface plasmon resonance experiments, coupled with alanine-scanning mutagenesis, revealed that two class II motifs, but not a class I motif, in p140Cap facilitated binding. Confocal microscopy and proximity ligation assays confirmed that BIN1 colocalizes with, and is within molecular distance of, p140Cap in cultured cells and in the mouse brain. Coimmunoprecipitation assays validated the interaction and glutathione S-transferase pulldown revealed that a rare BIN1 coding variant (*rs138047593*) significantly reduces p140Cap and tau binding, highlighting the impact of this mutant on interacting protein binding efficiency. The functional implications of BIN1:p140Cap interaction for neuronal functions warrant further investigation.

Bridging integrator 1 (*BIN1*), also known as Amphiphysin 2 or SH3P9, was originally cloned as a c-Myc–interacting protein (1). It was identified by genome-wide association studies (GWAS) as the second leading genetic risk factor for late-onset Alzheimer’s disease (AD) development after *APOE* (2, 3, 4, 5, 6). Most AD-associated *BIN1* variants are located upstream of the coding region and do not modify the protein sequence or structure but are hypothesized to increase AD risk by modulating gene expression (7, 8, 9). AD-associated *BIN1* SNPs are thought to alter cellular BIN1 expression, notably through a microglia-specific enhancer (7); however, the exact mechanisms by which *BIN1* SNPs participate in late-onset AD pathogenesis are only beginning to emerge. Expression quantitative trait loci colocalize with *BIN1* GWAS risk variants in human brain microglia and are associated with increased *BIN1* expression in a disease context (8). The index *BIN*1 rs744373 risk allele was associated in older adults with increased cerebrospinal fluid tau levels and greater brain tau-PET accumulation in individuals with higher amyloid burden and cognitive decline (9, 10, 11), suggesting that *BIN1* late-onset AD variants increase the risk for AD by influencing tau pathology. Supporting evidence includes BIN1’s interaction with tau (12), neuronal BIN1’s influence on cell-to-cell tau spread and pathogenesis (13, 14), and a potential involvement of microglial BIN1 in promoting tau spreading (15). Interestingly, the rare *rs138047593 BIN1* coding variant (K385R)*,* which results in the substitution of a lysine for an arginine at position 30 within the Src homology 3 (SH3) domain, has been implicated in amyloid pathology (16). The impacts of this rare coding variant on tau pathology, however, have yet to be explored. While a considerable amount of research has been devoted to understanding the role of *BIN1* in AD pathophysiology, fundamental gaps in knowledge persist concerning BIN1’s precise function in neurons.

BIN1 is a member of the Bin1-Amphiphysin-Rvs (BAR) domain superfamily of adaptor proteins that participates in various aspects of membrane remodeling, including endocytosis, sensing, and imposing membrane curvature, and actin-cytoskeletal regulation (17, 18, 19). Through alternative splicing, *BIN1* generates multiple tissue- and cell type–specific, as well as ubiquitous, isoforms that maintain conserved N-terminal BAR and C-terminal SH3 domains with a variable central region (20, 21, 22, 23). BIN1 is highly expressed in skeletal muscle and the brain. In the brain, BIN1 is predominantly expressed in the white matter tracts and mature oligodendrocytes; however, BIN1 is also expressed in neurons and microglia (23, 24). We previously reported that neuronal BIN1 localizes to presynaptic and postsynaptic compartments in excitatory neurons, where conditional knockout of BIN1 expression significantly increased the reserve pools of synaptic vesicles and revealed a deficit in neurotransmitter vesicle release (25). In agreement, neuronal BIN1 has been correlated with excitability in various cell and animal models (26, 27, 28). While we and others have demonstrated a role for BIN1 in synaptic transmission, details outlining the molecular mechanism remain elusive.

The C terminus of BIN1 possesses an invariant SH3 domain across all isoforms. SH3 domains in proteins promote transient protein–protein interactions that facilitate cell signaling and regulation, as well as the assembly of macromolecular complexes (29, 30). Most SH3 domains bind with an equilibrium dissociation constant (K_D_) ranging from 1 to 100 μM for the rapid association and dissolution of multiprotein complexes (31). Peptides from dynamin, an essential protein in endocytosis, have produced the strongest cellular BIN1 SH3-mediated ligand affinity constants to date, with K_D_ = 8 to 20 μM (32). Strikingly, a peptide from the Chikungunya virus nsP3 protein has been demonstrated to bind BIN1’s SH3 domain with an unusually high affinity of K_D_ = 24 nM (32). A recent study identified significant interactions between the BIN1-SH3 domain and 188 full-length cellular proteins in Jurkat cell extracts utilizing a novel approach termed native holdup, and reported a K_D_ range from 0.5 to 34 μM (33). Additionally, several biophysical studies have reported a direct interaction between BIN1’s SH3 domain and a class II proline-rich motif (PRM) on tau [P^216^xxPxR^221^], thereby implicating BIN1 in tau pathology (34, 35, 36, 37). Reports concerning the affinity of this interaction range from 12 to 44 μM; however, binding is negatively regulated by a combinatorial effect of tau phosphorylation status observed in AD pathophysiology (34, 35, 36, 38).

The binding of targets to BIN1’s SH3 domain occurs through a collection of three proximal grooves: two independent xP dipeptide binding pockets, formed by hydrophobic interactions among conserved aromatic residues in the RT and n-Src loops, and an adjacent acidic pocket referred to as the specificity zone (39). The SH3 minimum consensus core, “PxxP,” adopts a left-handed polyproline-II helix that binds in one of two orientations: class I “+xxPxxP” and class II “xPxxPx+,” where “x” represents any residue and “+” represents a basic residue—typically arginine (40). Several SH3 domains exhibit atypical binding, which maintains dependence on hydrophobic prolines but does not adhere to the canonical motif framework, and a growing number demonstrate noncanonical binding, notwithstanding the presence of prolines (41). Variability in the sequence length and composition of the RT and n-Src loops alters the hydrophobicity of the xP binding pockets, providing SH3 domains with specificity for their corresponding ligands (42). Notably, the crystal structure of the rat BIN1-SH3 domain revealed two largely acidic inserts in the distal and n-Src loops that enhance the negative electrostatic potential and reduce the hydrophobicity of the binding pockets, thereby enabling BIN1 to engage noncanonical targets (39). Accordingly, the SH3 domain in the muscle-specific BIN1 isoform 8 has been reported to intramolecularly interact with a noncanonical “RxxK” motif within a phosphoinositide-interacting domain, encoded by the muscle-specific exon (43). However, intermolecular noncanonical binding of the neuronal BIN1 isoform has not been demonstrated. Overall, the preference of neuronal BIN1’s SH3 domain for ligands harboring a class II PRM has been evidenced in dynamin, synaptojanin, tau, RIN3, c-myc, several viral peptides, and intramolecularly within BIN1’s clathrin and AP-2–binding domain (32, 35). The abundance of PRMs in the human proteome, combined with the broad recognition potential and dynamic nature of SH3 domains, suggests that this list is incomplete. Extending the BIN1 SH3 domain interactome is a valuable first step in deciphering the function of BIN1 in various physiological and pathological processes, including cytoskeletal regulation, synaptic transmission, tau pathogenesis, and AD pathophysiology.

Here, we report p140Cap, encoded by the gene SRC kinase signaling inhibitor 1 (44), as a BIN1-SH3 domain binding partner in neurons. We demonstrate that the affinity of this interaction is biologically relevant through comparison with tau, a well-known neuronal BIN1-SH3 domain binding partner with moderate affinity. Through alanine-scanning mutagenesis, we provide evidence that the interaction between BIN1 and p140Cap is facilitated by a combination of two canonical class II PRMs. Immunofluorescence staining experiments confirm the interaction and establish that the binding of full-length neuronal BIN1 to p140Cap is mediated by the SH3 domain. Remarkably, we demonstrate a decrease in tau and p140Cap binding to the BIN1 SH3 domain harboring the rare coding variant K358R. Together, these results reveal p140Cap as a biologically relevant neuronal BIN1 binding partner and illuminate a new avenue for investigating native BIN1 function in neuronal processes that may provide insights into the mechanism of BIN1 in AD pathophysiology.

## Results

### A peptide from the p140Cap adaptor protein binds to BIN1’s SH3 domain

**Figure 1:**
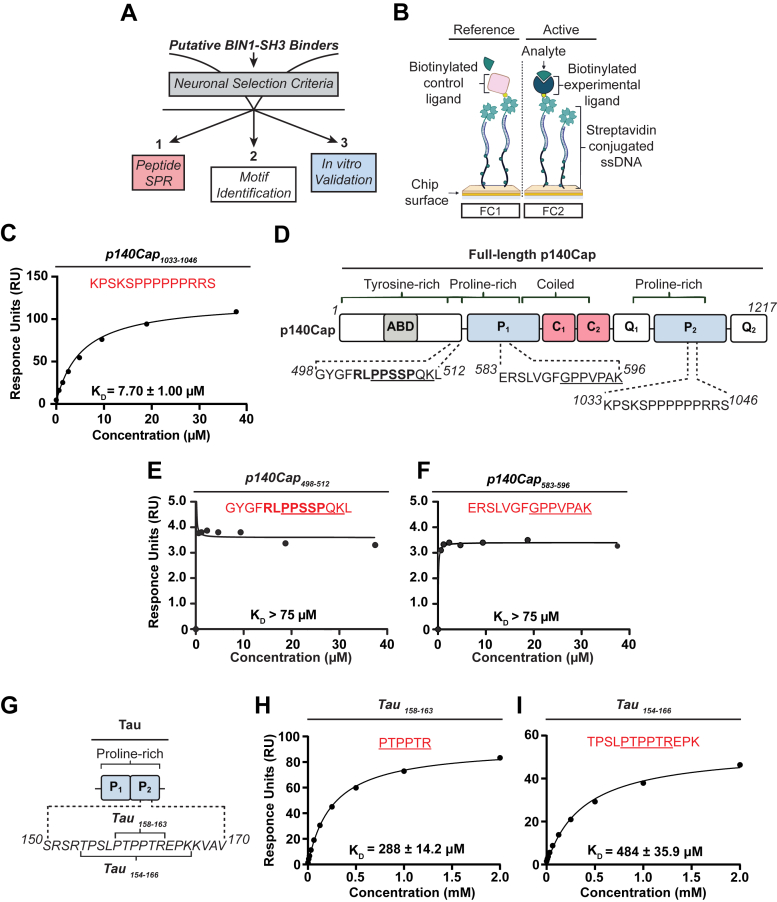
**SPR analysis of BIN1’s SH3 domain interactions with p140Cap and tau.***A*, schematic representation of the criteria used to select putative neuronal BIN1-SH3 domain–interacting proteins. *B*, schematic of the SPR experimental design using the Cytiva Biotin Capture chip. *C*, equilibrium analysis of the binding of 14-mer p140Cap_1033-1046_ to biotinylated BIN1-SH3. *D,* schematic of full-length p140Cap and each 14-mer peptide assessed by SPR. Class I motifs are shown in *bold* and class II motifs are shown *underlined*. *E-F*, equilibrium analysis of the binding of peptides 14-mer p140Cap_498-512_, and p140Cap_583-596_, respectively, to biotinylated BIN1-SH3 domain. *G*, sequences of the two peptides from the proline-rich domain of tau assessed by SPR. *H* and *I*, equilibrium analysis of the binding of 6-mer tau_158-163_ and 13-mer tau_154-166_ peptides to biotinylated BIN1-SH3 domain, with class II motifs *underlined*. Shown are representative plots averaged from three technical replicates, and the data are represented as mean ± SD. BIN1, bridging integrator 1; SH3, Src homology 3; SPR, surface plasmon resonance.

We undertook a candidate approach to identify biologically relevant neuronal proteins that interact with BIN1’s SH3 domain (Fig. 1*A*). First, we referenced published K_D_ calculations for several BIN1 SH3 domain ligands (45), setting a K_D_ cut-off at <10 μM for our study. Then, we obtained a published index of potential protein interactors from a study that computationally predicted the top 100 peptide binding partners for each of 70 human SH3 domains, generated by SH3PepInt, an alignment-free, graph kernel-based tool (46). We curated the predicted BIN1 SH3-binding proteins using a set of criteria to ensure: 1) neuronal expression, 2) subcellular localization at the synapse, and 3) molecular functions relevant to neuronal BIN1 in neurotransmitter vesicle dynamics (25). We then utilized available protein structures in the Protein Data Bank or those predicted by AlphaFold to confirm the accessibility of each putative SH3-binding site within the full-length protein, prioritizing peptides known to adopt the left-handed polyproline-II helix. From these analyses, we nominated 10 proteins for an initial screening using 14-mer peptides in surface plasmon resonance (SPR) analysis (Fig. 1*B*). The purified Avi-tagged BIN1 SH3 domain was immobilized as the ligand on flow cell 2, while a nonbinding control protein was immobilized on flow cell 1 (Fig. S1). Subsequently, each peptide was injected in solution over the sensor surface as the analyte (Fig. 1*B*). To correct for nonspecific binding, response units from flow cell 1 were subtracted from those of flow cell 2 to determine the K_D_.

The SPR screen identified a positive hit with a K_D_ of 7.70 ± 1.00 μM in a peptide, ^1033^KPSKSPPPPPPRRS^1046^, located within the C-terminal proline-rich domain of p140Cap (Fig. 1*C*). p140Cap, encoded by SRC kinase signaling inhibitor 1, was initially identified as a Src kinase signaling inhibitor (47). In neurons, p140Cap localizes to presynaptic and postsynaptic sites, and interacts with presynaptic proteins SNAP-25 and synaptophysin (48, 49). Examination of the human p140Cap sequence revealed three additional canonical PRMs within the N-terminal proline-rich domain; a class I (bold) and class II (underlined), ^498^GYGF**RLPPSSP**QKL^512^, and a single class II motif (underlined), ^583^ERSLVGFGPPVPAK^596^ (Fig. 1*D*). While both sequences are located within predicted disordered regions and are therefore theoretically available for SH3 binding, neither peptide, ^498^GYGFRLPPSSPQKL^512^ nor ^583^ERSLVGFGPPVPAK^596^, bound within the range of tested analyte concentrations (Fig. 1, *E* and *F*). In comparison, we tested a 6-mer and a 13-mer tau peptide harboring either the minimum consensus class II PRM alone, ^158^PTPPTR^163^, or extended C and N terminally, ^154^TPSLPTPPTREPK^166^ (Fig. 1*G*). While it is standard practice to test the analyte concentrations ranging from 0.1 to 10× the expected K_D_, we anticipated these peptides would bind weaker than the published K_D_ values due to their reduced length. Therefore, we extended the range of tested analyte concentrations accordingly. Both shorter and longer peptides exhibited highly transient affinities, (K_D_ = 288 ± 14.2 μM and 484 ± 35.9 μM, respectively) (Fig. 1, *H* and *I*), compared to the 12 to 44 μM observed from a 30-mer tau peptide in NMR and isothermal calorimetry experiments (35, 36, 37).

### The BIN1-SH3 domain recognizes a synergy of two class II PRMs on p140Cap

**Figure 2:**
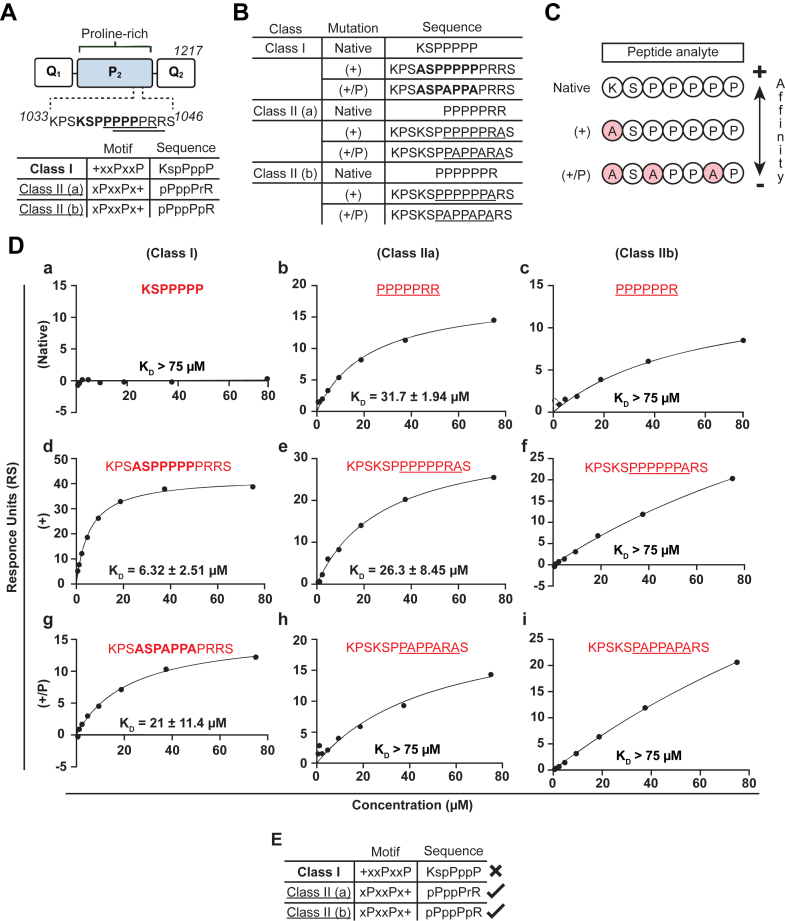
***SPR analysis of the BIN1–SH3 interaction with class II PRMs on p140Cap.****A*, schematic representation of the C-terminal proline-rich domain of p140Cap and the three canonical PRMs found within the 14-mer peptide sequence, where the class I motif is shown in *bold* and class II motifs are shown *underlined*. *B*, sequences of the 7-mer native and 14-mer alanine-substituted peptides synthesized for equilibrium assessment by SPR. *C*, schematic representation of the alanine-scanning mutagenesis experimental design and anticipated outcomes, where substitution of the basic residue alone or in combination with the corresponding proline residues is expected to result in decreased binding strength compared to the native 7-mer peptide. *D*, equilibrium analysis of the binding of the indicated peptides to biotinylated BIN1-SH3 domain; *a-c*, native class I, class IIa, and class IIb 7-mer p140Cap peptides, respectively; *d-f*, the corresponding 14-mer peptides containing alanine-substitution of the basic residue; *g-i*, the corresponding 14-mer peptides containing alanine-substitution of the basic residue and two corresponding proline residues. Shown are representative plots averaged from three technical replicates, and the data are represented as mean ± SD. *E*, summary of the mutagenesis/SPR results. BIN1, bridging integrator 1; SH3, Src homology 3; SPR, surface plasmon resonance.

The ^1033^KPSKSPPPPPPRRS^1046^ p140Cap peptide harbors three canonical PRMs: class I, ^1036^**KSPPPPP**^1042^; class IIa, ^1038^PPPPPRR^1045^; and class IIb, ^1037^PPPPPPR^1044^ (Fig. 2*A*). While it is generally accepted that BIN1’s SH3 domain displays a preference for class II motifs, we sought to exclude the possibility of a class I–mediated interaction. Thus, we used SPR in combination with alanine-scanning mutagenesis to determine which of the three class motifs on p140Cap mediates binding to BIN1’s SH3 domain. We synthesized 14-mer peptides with alanine substitutions at either the basic residues alone or in combination with the corresponding prolines for all three canonical PRM classes. We also synthesized 7-mer peptides containing the native core motif for cross-referencing (Fig. 2*B*). SPR affinity analyses were performed by testing each of the 9 peptides in concentrations ranging ∼10-fold above and below our cut-off of 7.70 μM (0.6–75 μM) to assess their K_D_s relative to the original 14-mer peptide ^1033^KPSKSPPPPPPRRS^1046^. We reasoned that alanine substitution of residues not involved in BIN1-SH3 binding would produce similar, if not identical, K_D_s to the native 14-mer peptide. Conversely, substitution of residues that most contribute to binding would yield significantly weaker K_D_s to BIN1-SH3 (Fig. 2*C*). Thus, we would be able to identify the precise motif responsible for facilitating the BIN1–p140Cap interaction.

In accordance with the principle that BIN1’s SH3 domain prefers class II PRMs, the 7-mer minimum consensus class I motif exhibited no binding within the range of tested analyte concentrations (Fig. 2*Da)*. Subsequent alanine substitution of the lysine in the 14-mer peptide had no significant impact on BIN1-SH3 domain affinity (K_D_ = 6.32 ± 2.51 μM) compared to the native 14-mer peptide (Fig. 2*Dd*); however, substitution of the lysine in combination with its corresponding prolines decrease affinity ∼3.3-fold (K_D_ = 21.0 ± 11.4 μM) (Fig. 2*Dg*). Interestingly, the prolines within the class I motif align with those in the class IIb motif, suggesting that the prolines, rather than the lysine, contribute to BIN1-SH3 domain affinity. Next, the native class IIa 7-mer peptide yielded a ∼4-fold reduction in affinity compared to the native 14-mer peptide (K_D_ = 31.7 ± 1.94 μM) (Fig. 2*Db*). This observation suggests that residues outside the core motif may facilitate binding. Together with our previous alanine substitution results, these data suggest that the second proline of the polyproline region may be involved in binding to BIN1. Interestingly, compared with the original sequence, alanine substitution of the C-terminal arginine in the 14-mer class IIa peptide resulted in ∼3.4-fold decrease in binding affinity, (K_D_ = 26.3 ± 8.45 μM), thus demonstrating a moderate contribution of the C-terminal arginine to binding (Fig. 2*De*). Meanwhile, substitution of the arginine as well as its corresponding prolines resulted in a dramatic decrease in affinity that extended beyond 75 μM (Fig. 2*Dh*), confirming the importance of the prolines. These findings implicate the class IIa motif in binding and suggest a moderate role for electrostatic contributions within the core class motif that may not be as influential as previously thought. Finally, the native 7-mer class IIb peptide yielded a K_D_ outside the range of tested analyte concentrations (Fig. 2*Dc*). Subsequent substitution of the N-terminal arginine in the 14-mer peptide alone or in combination with its corresponding prolines had similar impacts on affinity (Fig. 2, *Df and Di*). Comparing the two arginine mutations (Fig. 2, *De versus Df*) suggests that the N-terminal arginine may contribute more to binding than the C-terminal arginine, these results implicate the arginine and corresponding proline residues in the class IIb motif in binding. Based on the above results, we conclude that BIN1’s SH3 domain does not utilize the class I motif to mediate binding. Moreover, a synergy between the class IIa and IIb motifs may support the binding affinity, with both the prolines and the two arginines all playing a role (Fig. 2*E*).

### BIN1 and p140Cap colocalize *in vitro*

**Figure 3:**
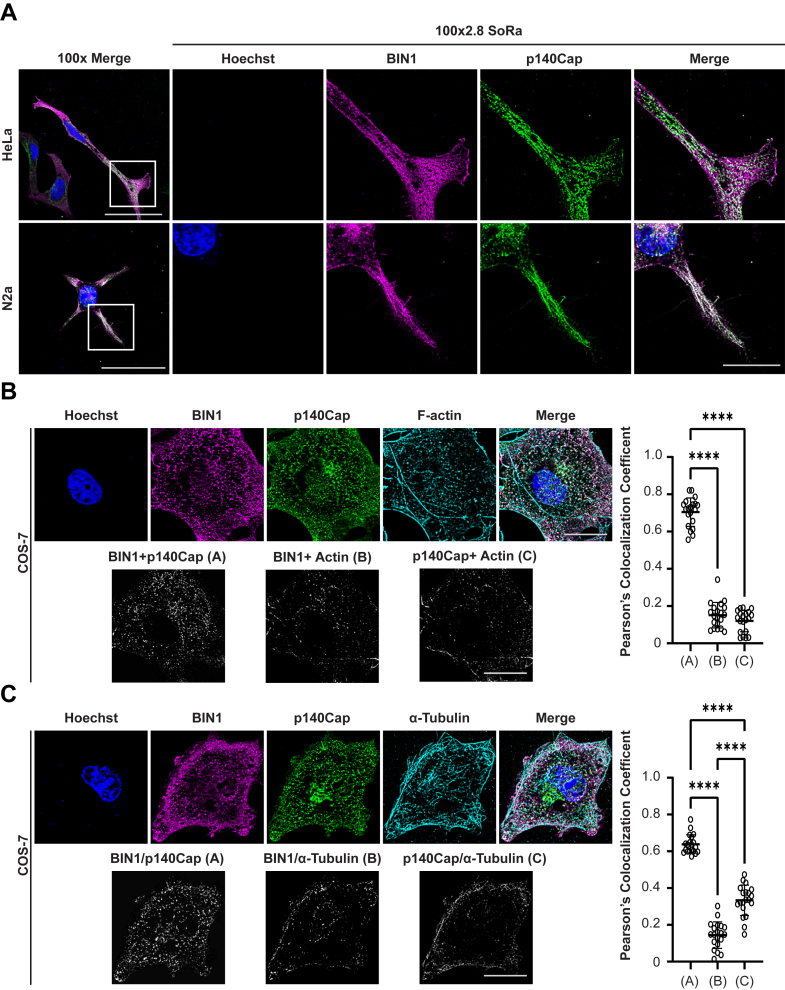
**Colocalization of neuronal BIN1 and p140Cap in cultured cells.***A*, HeLa (*top*) and N2a (*bottom*) cells were cotransfected with plasmids encoding neuronal BIN1 TID (*magenta*) and p140Cap-myc/His (*green*) and visualized by immunostaining. The colocalization between BIN1 and p140Cap (*white*) is represented in the merged images. 100X scale represents 50 μm, 100X 2.8 scale represents 20 μm. *B*, COS-7 cells were cotransfected with neuronal EGFP-BIN1 (pseudocolored *magenta*) and p140Cap-myc/His (*green*) and visualized by EGFP fluorescence and antibody staining, respectively, along with F-actin staining by Alexa Fluor 647–labeled phalloidin (*cyan*). Deconvolved confocal image stacks were used to calculate the degree of overlap in voxels between pairs of channels as Pearson’s colocalization coefficients (n = 20 cells from two transfections; results are plotted as mean ± SD). The *bottom gray-scale panels* depict the colocalization maps (*bottom*). One-way ANOVA revealed a significant difference between the three groups [F(2,57) = 467.8, *p <* 0.0001]. *C*, transfected cells were immunostained and analyzed as described above to ascertain the overlap between BIN1 (*magenta*) p140Cap (*green*) and α-tubulin (*cyan*). Deconvolved confocal image stacks were used to calculate the degree of overlap in voxels between pairs of channels as Pearson’s colocalization coefficients (n = 20 cells from two transfections; results are plotted as mean ± SD). One-way ANOVA indicated a significant difference between the three groups [F(2,57) = 254.3, *p <* 0.0001]. *Post hoc* Tukey’s analysis was performed. ∗∗∗∗*p <* 0.0001. BIN1, bridging integrator 1; SH3, Src homology 3.

To assess the subcellular localization of BIN1 and p140Cap, we performed immunofluorescence staining in mouse N2a neuroblastoma and HeLa cells cotransfected with expression plasmids encoding the human neuronal BIN1 isoform 1 and human p140Cap. HeLa cells have previously been used for characterizing neuronal Amph1 and BIN1 (50), and N2a cells were chosen because of their neuronal origin and characteristics. In both cell types, BIN1 was diffusely localized throughout the cytosol, within small punctate structures, the cytoskeleton, and along the plasma membrane (Fig. 3*A*). Consistent with previous studies, p140Cap localized to punctate vesicular and tubular structures in the cytosol and was enriched in the perinuclear area (49, 51). Interestingly, most of observed colocalization between BIN1 and p140Cap was found within membrane protrusions and filamentous patterns indicative of localization along the cytoskeleton (Fig. 3*A*). To further characterize the colocalization of BIN1 and p140Cap, African monkey kidney fibroblast-like COS-7 cells were transiently cotransfected with BIN1 and p140Cap, and immunofluorescence staining was performed to visualize either protein along with filamentous actin or α-tubulin. COS-7 cells were ideal for this experiment due to their flat morphology, which facilitated colocalization analysis. Image stacks were deconvolved, and Pearson’s colocalization coefficient was calculated to measure the degree of correlative variation of the two proteins across all image voxels. The results showed considerable colocalization between BIN1 and p140Cap (Pearson’s r = 0.70), compared to weak colocalization of either of the two proteins with F-actin (BIN1 r = 0.15 and p140Cap r = 0.12), (Fig. 3*B*). One-way ANOVA revealed a significant difference between the three groups [F(2,57) = 467.8, *p <* 0.0001]. Staining of α-tubulin displayed similar colocalization between BIN1 and p140Cap, r = 0.64, compared to moderate colocalization of p140Cap with α-tubulin, r = 0.33, and weak colocalization of BIN1 with α-tubulin, r = 0.14 (Fig. 3*C*). One-way ANOVA indicated a significant difference between the three groups [F(2,57) = 254.3, *p <* 0.0001]. Together, these results indicate that BIN1 and p140Cap colocalize in cultured cells, and their colocalization does not necessarily involve association with the cytoskeleton.

**Figure 4:**
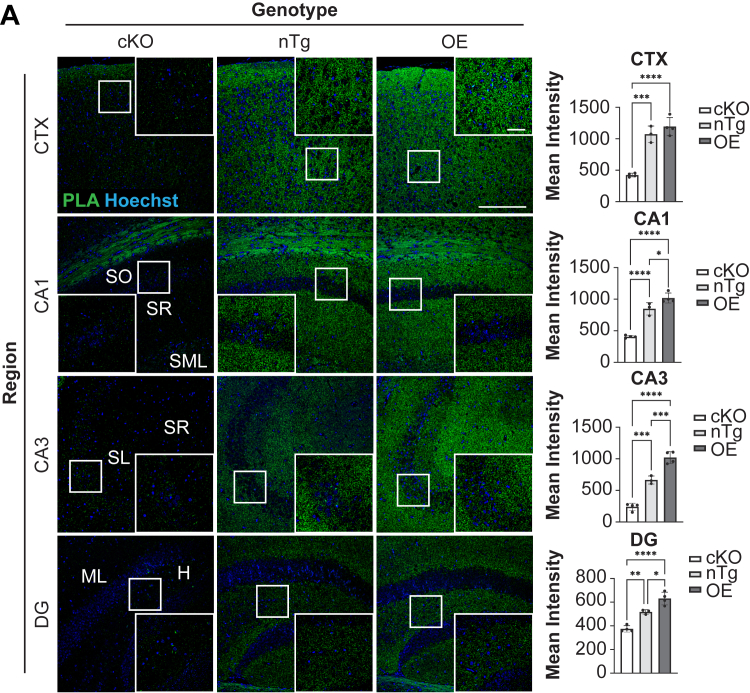
**PLA analysis of neuronal BIN1 and p140Cap in the mouse brain.***A*, representative images of PLA in the cortex (CTX) and hippocampal CA1, CA3, and dentate gyrus (DG) regions of the mouse brain. Images were acquired using a 20X objective (the scale bar represents 200 μm). The *boxed regions* are shown as a higher magnification image acquired using a 60X objective (the scale bar represents 50 μm). The PLA signal intensities from biological replicates of BIN1-cKO (n = 4), nTg (n = 3), and BIN1-OE (n = 4) animals were quantified, and the results are plotted as mean ± SD (*right*). The statistical significance was determined using ANOVA: CTX [F(2,8) = 54.39; *p <* 0.0001; CA1 [F(2, 8) = 79.76, *p* < 0.0001]; CA3 [F(2, 8) = 109.2, *p* < 0.0001]; DG [F(2, 8) = 46.00, *p* < 0.0001]. *Post hoc* Tukey’s analysis was performed. ∗*p <* 0.05; ∗∗*p <* 0.01; ∗∗∗*p <* 0.001; and ∗∗∗∗*p <* 0.0001. BIN1, bridging integrator 1; BIN1-OE, BIN1 overexpression; cKO, conditional KO; nTg, nontransgenic; PLA, proximity ligation assay; SH3, Src homology 3.

We then performed proximity ligation assays (PLAs) to confirm that BIN1 colocalizes with, and is within molecular distance (40 nm) of, p140Cap in the mouse brain. This *in situ* proximity approach allows for the detection and quantitative assessment of interactions between molecules with high sensitivity. We performed PLA using fixed tissue sections from nontransgenic (nTg) mice and compared the results with assays conducted in brain sections from conditional KO (cKO) mice lacking BIN1 expression in forebrain excitatory neurons and oligodendrocytes (BIN1-cKO) (25). Fixed brain tissue sections were stained using a BIN1 rabbit mAb and p140Cap mAb and processed for PLA. PLA signals were readily observed in nTg mice in the cortex and throughout the hippocampus (Fig. 4). As expected, the mean PLA fluorescence intensities were significantly lower in assays using BIN1-cKO tissue sections lacking forebrain BIN1 expression (Fig. 4, right). The residual PLA signal in BIN1-cKO mice likely indicates an interaction between BIN1 and p140Cap in inhibitory neurons and microglia, where BIN1 is still expressed in this model. Although BIN1 is abundantly expressed in mature oligodendrocytes and the white matter, the PLA signal over the corpus callosum is likely an artifact because BIN1 alleles are ablated in oligodendrocytes in BIN1-cKO mice and p140Cap is not expressed in the white matter (49, 52). To further extend the PLA approach, we prepared tissue sections from a transgenic mouse line, *B6 Tg(Bin1)U154.16.16Yah*, which overexpresses BIN1 (BIN1 overexpression [BIN1-OE]) from the human *BIN1* transgene (53), stained them using BIN1 and p140Cap antibodies, and processed them for PLA. BIN1/p140Cap PLA signals were readily observed in the cortex and throughout the hippocampus. One-way ANOVA analysis of the PLA signal intensities from the groups revealed significant differences among the three genotypes within the dentate gyrus, CA1, and CA3 of the hippocampus that correlated with BIN1 expression (Fig. 4). These results suggested that BIN1 and p140Cap are coexpressed in cortical and hippocampal neurons in the mouse brain, where they colocalize and likely interact.

### p140Cap expression increases in the CA3 hippocampal region upon BIN1-OE

**Figure 5:**
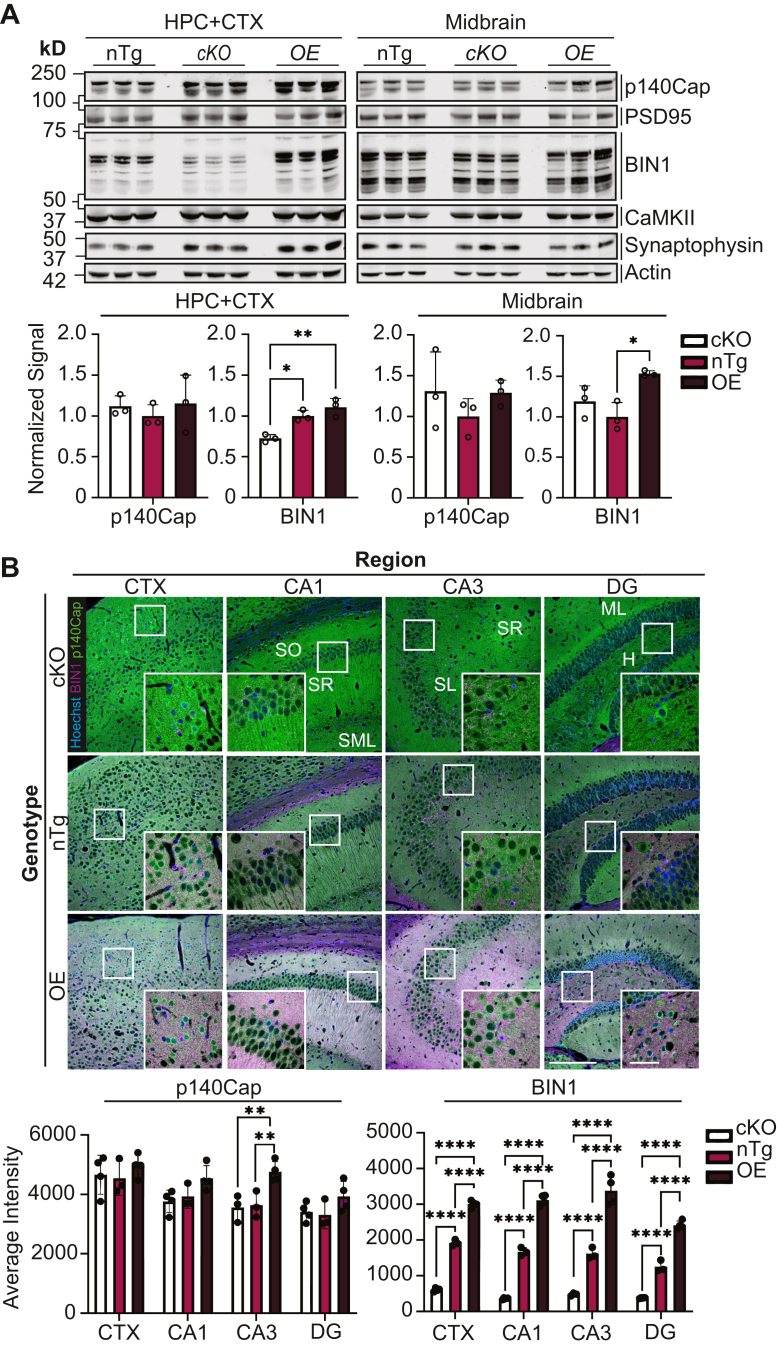
**Quantitative analysis of Bin1 and p140Cap expression in the brains of mice expressing varying levels of BIN1.***A*, immunoblot analysis of forebrain [hippocampus (HPC) and cortex (CTX)] or midbrain protein extracts from nTg, BIN1-cKO, and BIN1-OE animals (n = 3 mice per genotype). The blots were probed with antibodies against BIN1, p140Cap, PSD95, CaMKII, and synaptophysin. All bands observed in the blots represent distinct BIN1 isoforms [neuronal and ubiquitous isoforms (25, 52)], while p140Cap consistently appears as a doublet. ImageStudio 6.0 was used to quantify BIN1 and p140Cap signal intensities, and the results (normalized to nTg) are plotted as mean ± SD. One-way ANOVA found significant differences for BIN1 levels in HPC + CTX [F(2, 6) = 18.78, *p* = 0.0026] and midbrain [F(2, 6) = 9.345, *p* = 0.0144]; *post hoc* Tukey’s analysis was performed. ∗*p <* 0.05 and ∗∗*p <* 0.01. *B*, representative immunofluorescence images of mouse brains immunostained with antibodies against polyclonal BIN1 antibody (*magenta*) and mAb p140Cap (*green*). Images were acquired using a 20X objective from the cortex (CTX) and hippocampal CA1, CA3, and dentate gyrus (DG) regions (the scale bar represents 200 μm). The *boxed regions* are shown as a higher magnification image, acquired using a 60X objective (the scale bar represents 50 μm). BIN1 and p140Cap signal intensities were quantified from BIN1-cKO (n = 4 animals), nTg (n = 3 animals), and BIN1-OE (n = 4 animals), and the results are potted as mean ± SD (*right*). Two-way ANOVA revealed statistical differences in BIN1 [F(3,31) = 22.31, *p* < 0.0001] and p140Cap levels [F(2, 31) = 11.76, *p* < 0.001]. *Post hoc* Tukey’s analysis found significant differences in BIN1 levels in all regions (∗∗∗∗*p <* 0.0001) and p140Cap levels in CA3 (∗∗*p <* 0.01). BIN1, bridging integrator 1; BIN1-OE, BIN1 overexpression; cKO, conditional KO; nTg, nontransgenic; SH3, Src homology 3.

Next, we investigated whether p140Cap expression correlates with BIN1 expression levels in the mouse brain. To address this, we prepared homogenates from the forebrains (cortex and hippocampus) and midbrains of nTg mice, BIN1-cKO, and BIN1-OE mice. We conducted quantitative Western blot analyses, probing for BIN1 and p140Cap, in addition to presynaptic and postsynaptic markers. While BIN1 expression was quantitatively different among the three genotypes as expected, p140Cap expression did not differ (Fig. 5*A*).

We performed immunostaining with BIN1 and p140Cap antibodies to examine whether subtle subregional differences exist in the cortex and hippocampus (Fig. 5*B* and Fig. S5). As expected, two-way ANOVA showed that BIN1 levels differed significantly among the three genotypes in the cortex and hippocampal subregions examined [F(3,31) = 22.31, *p* < 0.0001] (Fig. 5*B*). Interestingly, we observed subtle differences in p140Cap levels across hippocampal subregions among the three genotypes. A two-way ANOVA indicated a significant effect of genotype on p140Cap levels [F(2, 31) = 11.76, *p* < 0.001]. Tukey *post hoc* tests found that p140Cap was significantly higher in BIN1-OE animals than in nTg or BIN1-cKO animals (*p <* 0.01) (Fig. 5*B*). Because the CA3 region contains a high density of axon terminals and synapses where BIN1 and p140Cap localize, the differences in p140Cap expression observed by immunofluorescence staining in this subregion was likely diluted by quantitative Western blot analysis of the entire forebrain tissue. From these results, we conclude that BIN1 expression has a subtle yet significant impact on p140Cap levels in specific synapse-rich areas of the mouse brain.

### BIN1-p140Cap binding is SH3 domain–mediated

**Figure 6:**
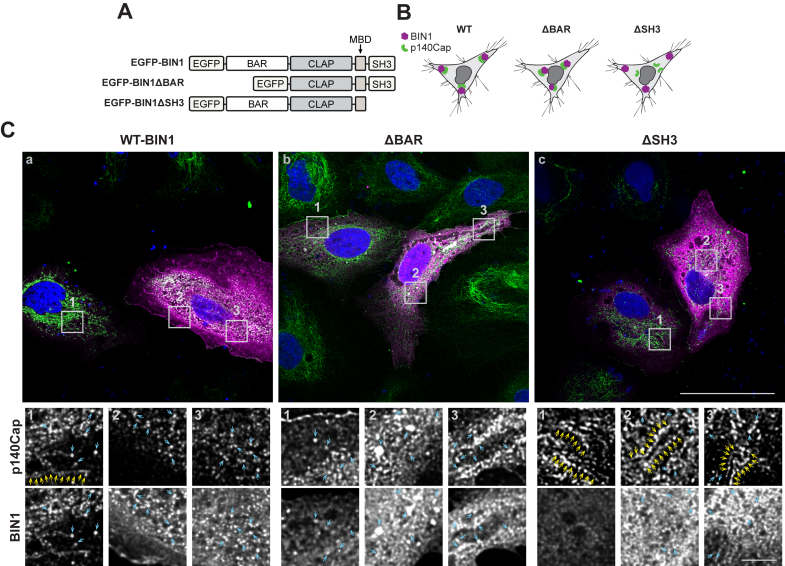
**BIN1-SH3 domain-dependent redistribution of p140Cap in HeLa cells.***A*, schematic representation of EGFP-tagged BIN1 constructs cotransfected with p140Cap-myc/His/His. *B*, *cartoons* depicting the anticipated experimental outcomes. *C*, HeLa cells cotransfected with p140Cap-myc/His and EGFP-tagged BIN1 WT (WT-BIN1), and BIN1 lacking the N-terminal BAR domain (ΔBAR) or the C-terminal SH3 domain (ΔSH3). Cells were immunostained to visualize p140Cap (*green*) and EGFP-BIN1 fluorescence (pseudocolored in *magenta*). 100X images scale represents 50 μm, and inset image scale represents 5 μm. The train of *yellow arrows* in the inset images indicates likely p140Cap localization along the cytoskeleton. *Blue arrows* indicate overlap in p140Cap and BIN1 localization. BAR, Bin1-Amphiphysin-Rvs; BIN1, bridging integrator 1; EGFP, enhanced green fluorescence protein; SH3, Src homology 3.

As BIN1 and p140Cap are multidomain proteins, we considered that multivalent interactions of BIN1, involving regions outside the SH3 domain could facilitate binding to p140Cap. To address this, HeLa cells were cotransfected with p140Cap-myc/His and one of three enhanced green fluorescence protein (EGFP)-tagged BIN1 constructs: full-length BIN1 (WT-BIN1), BIN1 lacking the N-terminal BAR domain (BIN1ΔBAR), or BIN1 lacking the C-terminal SH3 domain (BIN1ΔSH3) (Fig. 6*A*). We hypothesized that deletion of either domain may reveal a mislocalization of p140Cap within cells expressing the BIN1ΔBAR and ΔSH3 constructs (Fig. 6*B*). In nontransfected cells, the majority of p140Cap-positive puncta localized to the perinuclear space and also aligned along filamentous patterns (Fig. 6*C a, inset 1*). In transfected cells, overexpressed full-length BIN1 signal was detected throughout the cytosol and along the plasma membrane. In these cells, p140Cap adopted a punctate staining pattern dispersed in the cytosol, with a subset localizing at plasma membrane ruffles. Notably, WT BIN1 expression appeared to alter p140Cap’s filamentous appearance and induced a more punctate cytosolic localization (Fig. 6*Ca, insets 2 and 3)*. In cells expressing BIN1ΔBAR, BIN1 localization at the plasma membrane was reduced, and instead, the deletion mutant adopted a dense cytosolic localization (Fig. 6*Cb, insets 2 and 3)*. In these cells, p140Cap also relocated to associate with BIN1 in larger amorphic and smaller punctate structures. In comparison, in adjacent cells with minimal BIN1ΔBAR expression, p140Cap localization was observed in a perinuclear and filamentous pattern and along the plasma membrane (Fig. 6*Cb, inset 1*). In cells expressing BIN1ΔSH3, the BIN1 localization appeared similar to that of full-length BIN1. Notably, p140Cap localization in cells expressing BIN1ΔSH3 was identical to that of an adjacent cell lacking BIN1 expression, suggesting that p140Cap localization is impervious to BIN1 lacking the SH3 domain (Fig. 6*Cc, insets 1–3*). These results indicate that the interaction between BIN1 and p140Cap is largely mediated by the SH3 domain and suggest that this interaction between the two proteins can alter p140Cap subcellular location.

### BIN1’s SH3 domain captures more p140Cap than tau from brain extracts

**Figure 7:**
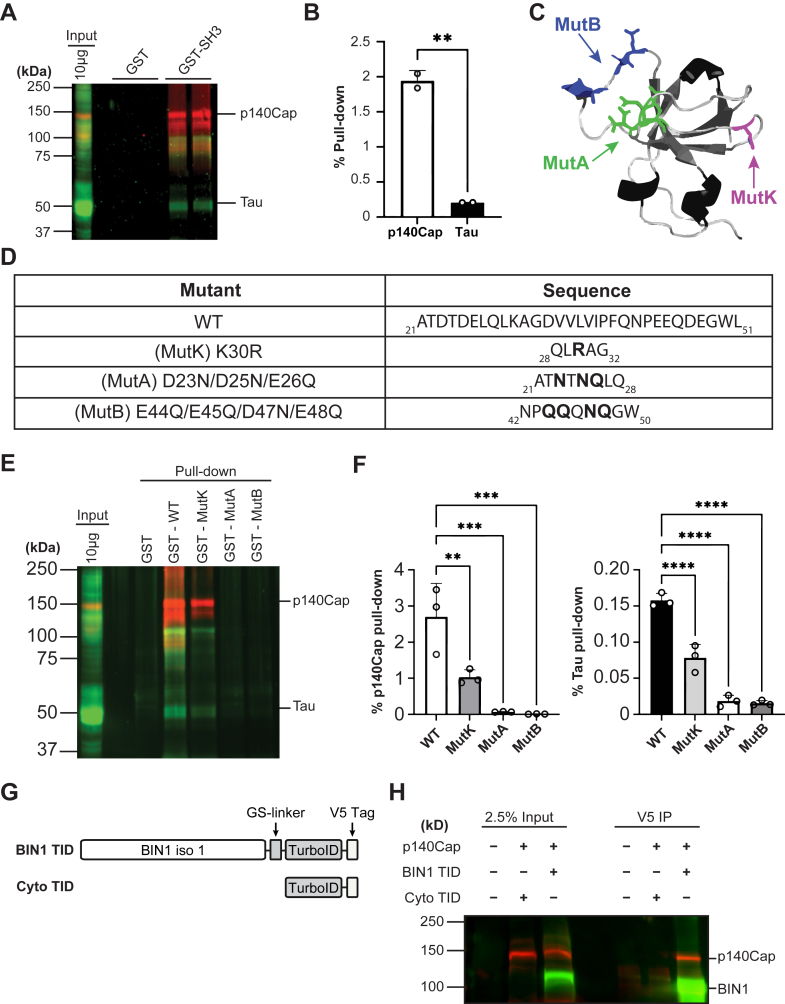
**GST pull-down analysis of BIN1-SH3 domain interaction with p140Cap and tau.***A*, GST pull-down assay of BIN1-SH3 binding to p140Cap and tau from mouse brain lysates. Mouse brains were homogenized in Hepes buffer, and 0.5 mg aliquots of 1% Triton X-100 soluble proteins were incubated with 25 μg of GST or GST-BSH3 coupled to glutathione-Sepharose. Bound proteins were analyzed by immunoblotting with antibodies against total tau and p140Cap. *B*, quantification of the efficiency of GST pulldowns. Immunoblots were analyzed on the LI-COR system, and the signal intensities from two independent experiments were quantified using ImageStudio 6.0. The efficiency of GST pulldown was calculated by comparing signal intensities of p140Cap or tau in the pulldowns with the corresponding signal in the input lane. The results are plotted as mean ± SD, and statistical significance was determined using an unpaired two-tailed *t* test; ∗∗*p =* 0.0035. *C*, Richardson diagram of the rat BIN1-SH3 domain crystal structure (PDB: 1BB9). Acidic residues mutated to their nonacidic counterparts in constructs used in GST pull-down experiments are represented by *sticks*; *blue* = MutB, *green* = MutA, *magenta* = MutK (K42R GWAS SNP). *D*, table outlining the mutations introduced into each construct. *E*, GST pulldowns from mouse brain homogenates using WT, BIN1-SH3, MutK, MutA, and MutB were conducted in triplicate as described above. *F*, bar chart from three technical replicates displaying the efficiency of GST pulldown of tau and p140Cap from each construct used. The pull-down efficiency was calculated as described above, and the results are plotted as mean ± SD. Statistical significance was analyzed by one-way ANOVA: p140Cap pulldown [F(3, 8) = 20.88, *p* = 0.0004]; tau pulldown [F(3, 8) = 104.0, *p* = 0.0001]. *Post hoc* Dunnett's analysis was performed. ∗∗*p <* 0.01; ∗∗∗*p <* 0.001; and ∗∗∗∗*p <* 0.0001. *G,* schematic representation of TurboID constructs cotransfected with p140Cap-myc/His. *H*, coimmunoprecipitation analysis of BIN1 and p140Cap. Subconfluent cultures of N2a-p140Cap cells grown on 60 mm dishes were transfected with plasmids encoding V5-tagged BIN1 TID or V5-tagged Cyto TID. The cells were lysed in 400 μl of co-IP buffer supplemented with 1% IGEPAL and incubated with 3 μl of mouse anti-V5 mAb. The resulting immunoprecipitates and input lysates, corresponding to 2.5% of the volume used for immunoprecipitation, were probed with antibodies against BIN1 and p140Cap. BIN1, bridging integrator 1; GST, glutathione S-transferase; GWAS, genome-wide association studies; SH3, Src homology 3.

To further validate the interaction between BIN1 and p140Cap *in* vitro, we conducted pull-down assays using mouse brain homogenate under conditions previously employed to demonstrate SH3–domain interactions among biologically relevant proteins (54, 55). For these experiments, we prepared glutathione S-transferase (GST) control and GST-tagged BIN1-SH3 domain fusion proteins coupled to glutathione-Sepharose and incubated them with mouse brain homogenates to capture bound proteins. Western blot analysis confirmed that p140Cap from the brain extract bound specifically to the BIN1-SH3 fusion protein, but not to the GST control (Fig. 7*A*). We probed the blots with tau antibodies and found that mouse brain tau was also isolated in this pull-down experiment. Interestingly, a comparison of the p140Cap and tau pull-down signal intensities to those in an aliquot of the input homogenate indicated that p140Cap was isolated by the BIN1-SH3 domain with a relatively higher efficiency than tau. An unpaired *t* test comparing the percent pulldown of p140Cap and tau (relative to input) revealed a significant difference t(16.79) = 2, *p* = 0.0035, indicating that ∼9.4-fold more p140Cap was captured than tau (Fig. 7*B*). These results complement the data from our SPR analysis and suggest that BIN1’s SH3 domain has a higher affinity for p140Cap than tau (Fig. 1).

The results from our SPR assays using alanine-scanning mutants suggested that electrostatic interactions may not be as influential for BIN1 SH3 binding to its partners as previously thought. To further investigate the contributions of electrostatic interactions in BIN1 SH3 domain–mediated protein binding, we conducted GST pull-down assays with mutant BIN1 SH3 domain constructs that lacked charged residues. We generated two mutants by substituting pockets of acidic residues at the binding interface with their nonacidic counterparts: (MutA)—D23N/D25N/E26Q and (MutB)—E44Q/E45Q/D47N/E48Q (Fig. S2). Additionally, we generated MutK, which corresponds to a rare GWAS coding SNP (*rs138047593*), resulting in the substitution of a lysine for an arginine at position 30 of the SH3 domain to examine the impact of this SNP on p140Cap and tau binding (Fig. 7, *C* and *D*). Purified WT and mutant GST fusion proteins coupled to glutathione-Sepharose (Fig. S3) were incubated with mouse brain homogenates as described above. Proteins captured by the pulldown were probed for p140Cap and tau using Western blot analysis (Fig. 7*E*). Quantification of the immunoblot signals indicated that both acidic residue mutants significantly decreased tau and p140Cap binding to the BIN1-SH3 domain (Fig. 7*F*). These results contrast with our previous peptide mutagenesis findings, where the substitution of the basic residue in the class IIa motif produced a moderate yet, less than anticipated, impact on binding affinity (Fig. 2*De*). This discrepancy likely reflects an inherent protein–peptide asymmetry in the interaction, highlighting the greater structural and functional contribution of the SH3 domain residues. Intriguingly, we found that the K30R GWAS coding mutant bound ∼2-fold less tau and ∼2.6-fold less p140Cap compared to WT BIN1-SH3 (Fig. 7*F*). Nonetheless, these results confirm that electrostatic contributions play a significant role in BIN1 SH3 binding and underscore the impact of the *rs138047593* coding mutant on binding affinity.

Finally, we conducted coimmunoprecipitation (co-IP) assays to validate the interaction between full-length BIN1 and p140Cap coexpressed in N2a cells. Endogenous BIN1 and p140Cap levels in N2a cells were too low to be immunoprecipitated in this assay using the available antibodies. To overcome this limitation, we generated stable pools of N2a cells expressing p140Cap and confirmed p140 overexpression by Western blot and immunofluorescence cell staining (Fig. S4). Subsequently, V5-tagged neuronal BIN1 or a control protein was transiently expressed in stable p140Cap cells, and lysates were subjected to co-IP using V5 antibodies. Immunoblots showed the presence of p140Cap in immune complexes isolated from cells transfected with V5-tagged BIN1 and not the control protein, validating the interaction between BIN1 and p140Cap (Fig. 7*H*).

## Discussion

Although *BIN1* is recognized as a significant genetic risk factor for late-onset AD development, BIN1’s native function in neurons largely remains uncharacterized. We previously characterized BIN1’s essential role in presynaptic neurotransmitter vesicle release. However, the exact mechanism(s) remains unclear. Using a combination of biophysical and molecular approaches, we present the first experimental validation of p140Cap as a synaptic protein that interacts with BIN1, providing an initial characterization of this interaction *in vitro*, in cultured cells, and in the mouse brain. Our studies identified a peptide from the C-terminal proline-rich domain of p140Cap that binds to BIN1’s SH3 domain with an affinity (K_D_ = 7.7 μM) comparable to those of well-characterized and functionally relevant interactors, including dynamin and tau (K_D_ = 8–20 μM and 12–44 μM, respectively) (32, 34, 35, 36, 37). Using colocalization experiments, including immunofluorescence staining and PLA, we show that BIN1 and p140Cap colocalize within a molecular distance of 40 nm in the mouse brain. SPR experiments, coupled with alanine-scanning mutagenesis, showed that BIN1’s SH3 domain utilizes a combination of two canonical class II PRMs, but not a class I motif, to bind p140Cap. Mutagenesis experiments combined with GST pull-down assays demonstrate the significance of electrostatics in BIN1 SH3 domain–mediated interactions and provide evidence that BIN1 binds full-length p140Cap with higher affinity than its corresponding peptide. While this study was underway, we concurrently discovered through promiscuous biotin ligase–mediated biotinylation coupled with mass spectrometry that p140Cap is part of a cohort of neuronal BIN1-proximal proteins in N2a cells (56). These findings are highly relevant to the neuronal function of BIN1, as both BIN1 and p140Cap have been implicated in cytoskeletal regulation and synaptic transmission in neurons (25, 26, 51, 57, 58, 59, 60). p140Cap is highly expressed in the brain and has been shown to play roles in dendritic spine morphogenesis and synaptic plasticity (51, 57, 58, 61). Specifically, p140Cap has been localized to presynaptic and postsynaptic sites in excitatory synapses, similar to the synaptic localization of BIN1 we previously reported (25, 49).

While seeking to identify biologically relevant neuronal BIN1-SH3 domain binding proteins, we referenced the peptide affinity data for putative ligands derived from the high-density peptide array experiment and computational predictions (46). We then used SPR assays to identify a PRM peptide from the C-terminal proline-rich domain of p140Cap, which bound at K_D_ = 7.7 μM. To date, the synaptic protein ligand with the highest affinity to BIN1’s SH3 domain is a PRM found in dynamin 1 [K_D_ ranging from 8 to 20 μM (32)], a neuron-specific GTPase concentrated in presynaptic terminals where it plays key roles in synaptic vesicle endocytosis (62). In our GST pull-down assays using mouse brain homogenates, we observed a 9-fold increase in the amount of endogenous p140Cap bound to the BIN1-SH3 domain compared to tau. Additional validation using full-length proteins is required for comparative affinity assessments.

Several neuronal SH3 domain–containing proteins involved in cytoskeletal regulation can bind the C-terminal PRM on p140Cap (63). Moreover, p140Cap regulates actin dynamics in dendritic spines through complex mechanisms, including the inhibition of Src kinase and the binding to the scaffold protein Citron-N, the F-actin–binding protein cortactin, and the microtubule plus-end tracking protein EB3 (61, 64). BIN1’s role in cytoskeletal regulation is relatively obscure. An alternatively spliced cardiac BIN1 isoform has been shown to bind actin in the adult mouse heart and promote N-WASP–dependent actin polymerization (65). Whereas BIN1 has been shown to promote actin bundling *in vitro*, this activity is not abolished by mutants that disrupt BIN1’s BAR domain or truncate the SH3 domain (50, 59). Moreover, the neuronal BIN1 isoform 1 failed to associate with actin under conditions where actin association with BIN1 isoform 8 was readily observed (50). These findings raise ambiguity regarding which BIN1 domains and isoforms participate in actin regulation. Nevertheless, our studies cannot formally exclude a potential involvement of BIN1 in p140Cap regulation of actin dynamics in neuronal synapses.

In cultured cells, including HeLa cells, p140Cap localizes to perinuclear punctate structures and along filamentous structures reminiscent of the cytoskeleton (44, 49, 64). When cotransfected with BIN1, most of the p140Cap signal was found in perinuclear punctate organelles, with only a minor subset colocalizing with neuronal BIN1 isoform along filamentous processes. Like full-length BIN1, a mutant lacking the BAR domain also promoted the punctate cytosolic redistribution of p140Cap. This redistribution was not observed in cells coexpressing p140Cap and a BIN1 C-terminal deletion mutant that lacks the SH3 domain. Our experiments also revealed that when coexpressed, neuronal BIN1 and p140Cap showed minimal colocalization with F-actin visualized by phalloidin staining. Non-neuronal BIN1 isoforms have been reported to associate with microtubules in HeLa or COS-7 cells, likely through BIN1’s interaction with the microtubule plus-end tracking proteins CLIP-170 and EB3 (64, 66). However, we only observed minimal colocalization between α-tubulin and neuronal BIN1 in transfected COS-7 cells. We conclude that overexpression of BIN1 can influence p140Cap subcellular localization in an SH3 domain–dependent manner. However, detailed investigations on endogenous BIN1 and p140Cap in primary neurons may prove useful in elucidating the mechanistic relationship between the two proteins.

The successful outcome of the PLA experiment suggests that BIN1 and p140Cap localize within a molecular distance of 40 nm in the mouse brain. Although the PLA assay is highly sensitive, target-independent primary antibody binding can lead to false-positive signals due to this sensitivity. The inclusion of BIN1-cKO and BIN1-OE animals in our study facilitated the optimization and proper interpretation of our BIN1 and p140Cap PLA results. The absence of discernible signals within the neuronal soma, particularly within pyramidal neurons in the cortex and hippocampal regions, along with the overall punctate appearance of the PLA signals in the parenchyma indicates that the interaction between BIN1 and p140Cap may occur within neuronal synapses. Notably, the PLA signal was uniformly distributed across *stratum oriens*, *stratum radiatum*, and *stratum lacunosum-moleculare* in the CA1 region, *stratum lucidum* and *stratum lacunosum-moleculare* in the CA3 region, as well as the molecular layer and hilus of the dentate gyrus, indicating that the BIN1:p140Cap interaction likely occurs at both presynaptic and postsynaptic sites. Interestingly, we observed a potential spatial heterogeneity, marked by a significant increase in p140Cap levels in the hippocampal CA3 region of BIN1-OE animals. We believe this may reflect differential compartmentalization of p140Cap in areas with a high concentration of excitatory synapses.

The presence of three canonical PRMs within the C-terminal proline-rich domain of p140Cap, combined with the potential for multivalent interactions by various neuronal SH3 domain–containing proteins previously validated to bind the same sequence, led us to hypothesize that one motif may be specific to BIN1’s SH3 domain (63). While the majority of these p140Cap ligands (vinexin, cortactin, and p130Cas) utilize a class II PRM within the C-terminal proline-rich domain, co-IP, and GST pull-down experiments verified that endophilin A1 requires both N- and C-terminal proline-rich domains for binding (58). Additionally, Src has been shown to bind class I ligands, often with high affinity (63, 67). Although it is generally accepted that neuronal BIN1’s SH3 domain prefers a class II PRM, the limited list of experimentally validated binding partners prompted us to eliminate the possibility of a class I–mediated interaction. Results from our SPR assays combined with alanine scanning mutagenesis demonstrated that BIN1’s SH3 domain utilizes a combination of two class II PRMs, but not the class I motif, on p140Cap for binding. Specifically, we observed a dramatic decrease in affinity upon alanine substitution of the arginine and corresponding prolines in the class IIa motif, which resulted in a K_D_ above 75 μM. We noted a similar effect upon alanine substitution of the arginine alone, or in combination with the corresponding prolines, in the class IIb motif. We therefore conclude that a synergy between the class IIa and class IIb motifs mediates BIN1-p140Cap binding. Interestingly, we observed a ∼4-fold decrease in p140Cap’s affinity toward the BIN1 SH3 domain by alanine substitution of the C-terminal arginine from the class IIa motif alone. In comparison, an earlier study reported that alanine substitution of two lysine residues on tau reduced its affinity toward BIN1 by ∼10-fold (from ∼44 to ∼429 μM) (36), suggesting a more profound impact of electrostatics in the SH3 domain–mediated interaction between BIN1 and tau. While the canonical PxxP motif is important for SH3-mediated interactions, it is not always the sole determinant of binding. Previous studies have shown that additional residues, particularly basic ones flanking the xPxxPxR core, can significantly contribute to the overall interaction strength (30, 31, 40). Moreover, the inherent flexibility of peptides may allow them to tolerate certain substitutions without substantial loss of affinity. This is especially relevant when considering the class II motif tandem repeat in the 14-mer p140Cap peptide, which contains two adjacent arginine residues that may compensate for one another when one is mutated. In contrast, mutations in the SH3 domain, especially when targeting multiple acidic residues, disrupt both the structural and electrostatic features of the binding surface, thereby compromising peptide recognition and reducing binding affinity. It is worth noting that potential structural or avidity effects might be lost when using peptide-based assays. To overcome this limitation, we attempted to purify full-length p140Cap suitable for GST pulldown and SPR experiments. However, despite our efforts, purifying the full-length protein or even its N- and C-terminal halves was unsuccessful. These challenges underscore the need to validate relative affinities of both p140Cap and tau using full-length proteins. Nevertheless, when acidic residues at the SH3 binding interface were substituted with their nonacidic counterparts, we noted a significant reduction in both tau and p140Cap captured from mouse brain homogenates on Sepharose beads coupled to the BIN1-SH3 domain. These results suggest that the binding interaction is dependent on key residues within both the SH3 domain and the peptide, confirming the significance of electrostatic interactions in BIN1-SH3 domain binding to its interactors.

The rare AD GWAS coding region SNP *rs138047593*, which results in K358R substitution, has previously been shown to increase amyloid burden in the 5XFAD mouse model of amyloidosis (16). However, the impact of this coding mutant on tau binding propensity has not been studied until now. Interestingly, the *rs138047593* coding mutant, which causes the substitution of an arginine for a lysine at position 30 of the SH3 domain sequence (Figs. 7*C* and S2), captured tau from brain homogenates with significantly less efficiency in GST pull-down assays compared to the WT SH3 domain. Remarkably, this mutant also exhibited significantly less efficiency in capturing p140Cap than the native SH3 domain. We attribute this effect to the presence of the guanidinium group in arginine, which is inherently planar and ridged, potentially reducing the conformational flexibility required for optimal binding (68). Additionally, while it allows for increased hydrogen bonding compared to the lysine side chain at this position, its bulk may sterically interfere with specific protein interactions (68, 69). Since the residue (K30 or R30) is located outside the canonical SH3 domain binding interface, this observation suggests that this area may serve as a tertiary contact to mediate ligand binding.

In conclusion, our study demonstrates a direct protein–protein interaction between neuronal BIN1 and the scaffold protein p140Cap. Considering the synaptic localization of both proteins and their shared functional implications in cytoskeletal regulation and synaptic transmission, it is not surprising to observe a moderately high affinity between the two, compared to the highly transient, low-affinity binding typical of most SH3 domain–mediated interactions. The interaction between BIN1 and p140Cap in neurons may play a key role in synaptic biology by connecting membrane remodeling and neurotransmitter vesicle dynamics with actin cytoskeleton regulation (70, 71). BIN1 influences membrane curvature and scaffolding through its BAR and SH3 domains, whereas p140Cap impacts actin dynamics, particularly within dendritic spines, through interactions with cortactin and other actin-associated proteins. Together, they may contribute to the formation, maintenance, or stabilization of dendritic spines. Their interaction might also support the assembly of synaptic signaling complexes, thereby influencing synaptic plasticity. Furthermore, the BIN1–p140Cap interaction may affect presynaptic actin dynamics, which contributes to neurotransmitter vesicle docking, endocytosis, and maintaining neurotransmitter vesicle pool sizes in the presynaptic terminal. Disruptions in BIN1–p140Cap interactions could impair these processes and potentially lead to synaptic dysfunction, an early hallmark of AD. Nevertheless, understanding how this interaction is mechanistically relevant to neural function and whether it plays a role in AD pathophysiology are critical questions we plan to address in future studies. While large-scale approaches have identified putative interactors that bind to BIN1, only a few have been experimentally validated using orthogonal methods, particularly concerning the neuronal BIN1 isoform. This study presents the first report of p140Cap as a biologically relevant neuronal BIN1 binding partner and opens new avenues for functional investigations regarding native BIN1’s role in various physiological and pathological processes, including cytoskeletal regulation, synaptic transmission, and AD pathophysiology.

## Experimental procedures

### Animals

All experimental procedures related to animal care and experimental manipulation were approved by the Institutional Animal Care and Use Committee at the University of South Florida, Tampa. Mice were housed at 22 ± 2 °C under a 12 h light/dark cycle with *ad libitum* access to food and water. *Bin1*^*fl/fl*^ animals were generously provided by Dr George C. Prendergast (Lankenau Institute for Medical Research). *Emx1*-IRES-*Cre* (JAX stock #005628) lines were obtained from The Jackson Laboratory. *Emx*-Cre:*Bin1* conditional knockout mice were generated by crossing the *Bin1*^*fl/fl*^ strain with the *Emx1*-IRES-*Cre* driver line (25). Sperm to generate the BIN1 transgenic mouse line, *B6 Tg(Bin1)U154.16.16Yah*, was generously provided by Dr Yann Herault (IGBMC). This line has 5 to 10 copies of the entire *BIN1* locus and upstream sequences contained in a ∼195 kb human Bacterial Artificial Chromosome (53).

### Peptides

An index of the top 100 proteins predicted to bind to the BIN1 SH3 domain was obtained from a previous study (46). A subset of these predicted BIN1 SH3-binding proteins was then selected using specific criteria to ensure: 1) neuronal expression, 2) subcellular localization at the synapse, and 3) molecular functions relevant to neuronal BIN1 in neurotransmitter vesicle dynamics (25). Next, we used the software SH3PepInt [https://modpepint.informatik.uni-freiburg.de/SH3PepInt/Input.jsp*;* (72)] to identify 15-residue putative SH3-binding regions containing a combination of canonical “PxxP” and noncanonical “RxxK” motifs. We utilized available 3D structures from the Protein Data Bank or examined AlphaFold structural predictions to ensure SH3 domain accessibility in the corresponding full-length proteins. Putative interactors meeting these criteria were nominated for SPR analysis. Corresponding 14-residue peptides, based on the selected SH3-binding motifs, were synthesized by GenScript and purified *via* reversed-phase HPLC to ≥95% purity with N-terminal acetylation and C-terminal amination to remove free amide and carboxyl charges.

### Plasmid constructs

Sequences encoding the C-terminal 81 amino acids that encompass the SH3 domain of the human *BIN1* gene were synthesized (GenScript) and inserted into the pAviBir bacterial expression vector between BamHI and NotI restriction sites. The encoded fusion protein contains N-terminal His and Avi-tags and gets enzymatically biotinylated in *E.coli* upon expression within the 15 amino acid AviTag peptide (73). Similarly, sequences encoding the 263 amino acids that encompass the beta-lactamase of the bacterial *CTX-M-14* gene were inserted into the pAviBir bacterial expression vector between BamHI and HindIII sites. Sequences encoding the WT human BIN1 SH3 and those harboring substitutions, D23N/D25N/E26Q, E44Q/E45Q/D47N/E48Q, and K30R (the numbering corresponds to Gly residue 1 of the BIN1 SH3 domain), were synthesized and inserted into a His/GST bacterial expression vector as ScaI-NotI inserts to express N terminally 6xHis and GST-tagged BIN1-SH3 fusion proteins. pEF5/FRT NF plasmids encoding EGFP-tagged BIN1 constructs EGFP-BIN1, EGFP-BIN1ΔBAR, and EGFP-BIN1ΔSH3 were a kind gift from Dr Taisuke Tomita (The University of Tokyo) (74). The pcDNA plasmid encoding p140Cap-myc/His has been described (44). pLX304 plasmids encoding BIN1-TurboID-V5 (referred to as BIN1 TID) and cytosolic control (Cyto TID) were generated in the Thinakaran lab as described (56).

### Cell culture

HeLa and COS-7 cells were cultured in Dulbecco's modified Eagle's medium supplemented with 10% fetal bovine serum 100 U/ml penicillin and 100 μg/ml streptomycin at 37  °C in 5% CO_2_. Mouse N2a neuroblastoma cells were cultured in 50% OptiMEM and 45% Dulbecco's modified Eagle's mediumsupplemented with 5% fetal bovine serum, 1% Glutamax, 100 U/ml penicillin, and 100 μg/ml streptomycin, at 37  °C in 5% CO_2_. Cells grown on coverslips were transfected using 0.75 μg pcDNA p140Cap-myc/His (44) and 1 μg BIN1 plasmids (56, 74) using Lipofectamine 3000 (Invitrogen). N2a cells were transiently cotransfected with 2 μg pcDNA p140Cap-myc/His (44) and 100 ng of pMxPuro vector using Lipofectamine 3000. Stable pools were selected and maintained in 1 μg/ml puromycin and 0.4 mg/ml G418. The expression of p140Cap was confirmed by immunostaining and immunoblotting (Fig. S4).

### Protein expression and purification

All plasmids were transformed into the BL21 (DE3) bacterial strain. AviBir-M14 cells were grown in 1 L 2xYT medium [16 g tryptone, 10 g yeast extract, 5 g NaCl] supplemented with 0.2 M sorbitol and 5 mM betaine, and AviBir-Bin1 SH3 cells were grown in 1 L LB medium at 37 °C overnight. Prior to induction, 50 μM of biotin was added to the overnight culture. The expression of AviBir-M14 and BIN1 SH3 fusion protein was induced with 0.5 mM IPTG at 25 °C overnight. Bacterial cell pellets were resuspended in His buffer A [20 mM Tris (pH 8.0), 300 mM NaCl, 20 mM imidazole], supplemented with and a protease inhibitor cocktail (Thermo Fisher Scientific) and lysed by sonication using a 10 s/15 s on/rest cycle for 15 min at an amplitude of 6 to achieve near complete lysis. For purification, the lysate was centrifuged at 35,000g for 35 min at 4 °C, and the supernatant was filtered, and then loaded onto a HisTrap HP column (Cytiva Life Sciences) at 0.5 ml/min after sequential equilibration with His buffer B [20 mM Tris (pH 8.0), 300 mM NaCl, 500 mM Imidazole], His buffer A, and finally with water. The protein was eluted by a linear gradient (0%–100%) of His buffer B at 1 ml/min and the peak of the eluted protein was pooled and concentrated in Amicon Ultra centrifugal filters to approximately 3 ml in gel filtration buffer [10 mM Tris (pH 7.5), 50 mM NaCl, 2 mM EDTA]. The concentrated fusion protein was then loaded onto the HiPrep 16/60 Sephacryl S-200 HR gel filtration column (Cytiva Life Sciences). Peak fractions were pooled, concentrated in Amicon Ultra centrifugal filters to approximately 1 ml, and stored at −80 °C. Protein purity was evaluated by SDS-PAGE (Fig. S1).

GST fusion protein expression was induced with 1 mM IPTG and incubated for 3 h at 37 °C with vigorous shaking. Bacterial pellets were lysed in 1X PBS by sonication, followed by the removal of insoluble material through centrifugation at 42,000 rpm at 4 °C for 25 min. The clarified lysates were mixed on a nutator overnight at 4 °C with glutathione resin (GenScript) pre-equilibrated with 20 mM Tris (pH 7.5) containing 0.1% Triton X-100. The resin was washed four times with 20 mM Tris (pH 7.5) containing 0.1% Triton X-100, followed by a final wash with detergent-free 50 mM Tris (pH 8.0) and stored at −20 °C. Purity and concentration of the fusion proteins were assessed by SDS-PAGE gel electrophoresis, followed by Coomassie blue staining and bicinchroninic acid assays (Fig. S3).

### Surface plasmon resonance

All experiments were conducted using the Biacore S200 (GE Healthcare), equipped with a series S Biotin Capture chip (Cytiva Life Sciences) at 25 °C in 1X PBS-P + [0.2 M phosphate buffer with 27 mM KCl, 1.37 M NaCl, and 0.5% surfactant P20 (Tween 20)] running buffer. The chip surface was first conditioned with three 1-min injections of regeneration solution (3:1 8 M guanidine-HCl: 1 M NaOH) immediately after docking. Biotin CAPture reagent (50 μg/ml in HBS-EP buffer [0.01 M Hepes (pH 7.4), 0.15 M NaCl, 3 mM EDTA, 0.005% surfactant P20]), 3.4 ml) was immobilized on the chip surface at a flow rate of 2 μl/min for 5 min prior to ligand immobilization. Affinity run assays were conducted by immobilizing 50 μg/ml Avi-BSH3 (12 kDa, >90% pure based on SDS-PAGE) diluted in 1X PBS-P+ running buffer at 5 ul/min for 60 s on a streptavidin-coated matrix on flow cell 2. Additionally, 50 μg/ml Avi-tagged nonbinding protein (CTX-M-14 β-lactamase, 30 kDa, >90% pure based on SDS-PAGE) diluted in 1X PBS-P+ running buffer was immobilized at 5 μl/min for 60 s on flow cell 1 to serve as a reference surface for subtraction of bulk refractive index background. To collect affinity data, the analytes were diluted in running buffer and injected over the two flow cells in serial dilution at a flow rate of 30 μl/min. The complexes were allowed to associate and dissociate for 30 s and 300 s, respectively. The surfaces were then regenerated with three 60 s injections of regeneration solution. Triplicate injections of each sample and a running buffer blank were injected over the two surfaces. Data were collected at a rate of 40 Hz and fitted to a simple 1:1 interaction model using the steady-state analysis option available within the BIAevaluation 1.1 software (https://www.cytivalifesciences.com) to obtain equilibrium dissociation constants. Shown are representative plots averaged from three technical replicates and the data are represented as mean ± SD.

### Immunocytochemistry

Cells grown on coverslips were fixed in PBS containing 1 mM CaCl_2_, 1 mM MgCl_2_, and 4% paraformaldehyde at room temperature for 10 min, permeabilized with 0.2% Triton X-100 in PBS for 5 min at room temperature. The coverslips were blocked in 1X Dulbecco's phosphate-buffered saline (DPBS;Gibco) containing 3% bovine serum albumin (BSA), 50 mM NH_4_Cl, and 10 mM glycine for 15 min at room temperature, and then incubated with primary antibodies diluted in antibody dilution buffer (1X DPBS containing 0.2% Tween-20 and 3% BSA) overnight at 4 °C. Antibodies include polyclonal BIN1 antibody [BSH3, 1:500; validated in BIN1-cKO mice (25)], myc mAb 9e10 (1:250), and alpha-tubulin mAb (Sigma #T6074, 1:250). F-actin was stained using AlexaFluor 647–labeled phalloidin (Thermo Fisher Scientific #A22287, 1:40 dilution of a 66 μM stock). Cells were then washed in 1X DPBS containing 0.2% Tween-20 and incubated in Alexa Fluor–conjugated secondary antibodies diluted in antibody dilution buffer for 1 h at room temperature. After washing, cells were incubated in a 1:2500 dilution of Hoechst (5 mg/ml) for 5 min and mounted to glass slides using VECTASHIELD Antifade Mounting Medium.

### Tissue preparation and immunostaining

Mice were transcardially perfused with ice-cold 1X PBS. The brains were harvested and fixed overnight in 4% paraformaldehyde in 1X PBS and then preserved in 70% ethanol. Samples were embedded in paraffin, and 5 μM-thick coronal sections were prepared. The sections were cleared of paraffin in xylenes and rehydrated through submersion in increasingly diluted ethanol solutions. Epitope retrieval was performed by incubating sections in Reveal Decloaking solution (Biocare Medical) at 95 °C for 30 min using a Decloaking Chamber NxGen, followed by rinsing in 1X TBS (Biocare Medical). Nonspecific staining was blocked using Background Punisher (Biocare Medical) for 30 min. Sections were sequentially incubated with primary antibodies against p140Cap [Defilippi’s lab mAb 1:250 (44); validated in p140Cap KO mice (75)] for 48 h at 4 °C, and then BIN1 rabbit mAb [Abcam EPR13463-25, 1:1000; validated in BIN1-cKO mice (14)] for 1 h at room temperature. After incubating with Alexa Fluor–conjugated secondary antibodies, nuclei were counterstained with Hoechst 33342 before mounting the coverslips with VECTASHIELD Antifade Mounting Medium.

### Proximity ligation assay

Five micrometers thick coronal sections of nTg, BIN1-cKO, and BIN1-OE mouse brains were deparaffinized and subjected to antigen retrieval. The sections were then incubated with primary antibodies raised against BIN1 (Abcam EPR13463-25, 1:1000) and p140Cap (Defilippi’s lab mAb; 1:250) for 48 h at 4 °C. After washing, the sections were blocked with the Duolink Blocking Solution for 1 h at 37 °C. PLA was performed using the Duolink *in situ* fluorescence kit (Sigma) according to the manufacturer’s instructions. Coverslips were then applied with Duolink *in situ* mounting medium with 4',6-diamidino-2-phenylindole.

### Image acquisition and quantification

All images were acquired using an automated Nikon Ti2 microscope fitted with a Yokogawa spinning disc field scanning confocal system. Z-stacks of images were acquired using a 20X, 60X, or 100X objectives and were deconvolved with Huygens Professional v24.10 (https://svi.nl.Huygens-Professional) (Scientific Volume Imaging). For colocalization analysis, Pearson’s coefficient was calculated on the image z-stacks using Huygens Professional software. For quantitative analysis, immunofluorescence-stained brain images were imported into QuPATH and the average intensities for each channel were measured. The data for BIN1 and p140Cap staining were analyzed using a two-way ANOVA, whereas the PLA staining data were analyzed with one-way ANOVA in GraphPad Prism (https://www.graphpad.com).

### Immunoblotting

Hemibrain, forebrain, (hippocampus and cortex), and midbrain regions were weighed and homogenized (20% weight/volume) in ice-cold lysis buffer [150 mM NaCl, 50 mM Tris–HCl pH 7.4, 0.5% NP-40, 0.5% sodium deoxycholate, 5 mM EDTA, 0.25 mM PMSF, and protease inhibitor cocktail (Thermo Fisher Scientific). An equal volume of lysis buffer containing 2% SDS was then added to each sample and briefly sonicated. Aliquots of lysates were resolved by 4 to 20% polyacrylamide gel electrophoresis. Membranes were probed using antibodies against BIN1 (BSH3, 1:1000) (52), p140Cap (Defilippi’s lab mAb 1:2000) (44), synaptophysin (Sigma #S5768; 1:2000), PSD95 (Millipore # MABN68; 1:5000), CamKII (Santa Cruz Biotechnology # sc-32288; 1:500), and β-actin (Protein Tech #66009-1; 1:20,000). After incubation with infrared dye–conjugated secondary antibodies, signals were recorded using the Odyssey infrared imaging system (LI-CORbio) and quantified with ImageStudio6.0 (https://www.licorbio.com/image-studio).

### GST pull-down assay

We used a previously published method with some modifications (22). Mouse brains were homogenized at a ratio of 1:10 (w:v) in 20 mM Hepes, pH 7.4, containing a freshly added protease inhibitor cocktail and PMSF. Then, they were centrifuged for 5 min at 800*g*. The postnuclear supernatants were incubated with 1% Triton X-100 for 1 h at 4 °C, followed by centrifugation at 12,000*g* for 10 min to remove insoluble material. Aliquots of the soluble supernatant (0.5 mg protein) were incubated with 25 μg GST or GST-BSH3 coupled to glutathione-Sepharose (preblocked with 1 mg/ml BSA). After incubating for 2 h at room temperature, the beads were washed three times in 20 mM Hepes-KOH, pH 7.8, supplemented with 50 mM KCl, 150 mM NaCl, 1 mM EDTA, and 1.5% IGEPAL. Bound proteins were eluted by boiling at 95 °C for 5 min in Laemmli buffer. The samples were analyzed by immunoblotting with antibodies against total tau (Dako #A0024; 1:1000) and p140Cap (Defilippi’s lab mAb; 1:2000) (44). After incubation with infrared dye–conjugated secondary antibodies, signals were recorded using the Odyssey infrared imaging system (LI-COR Biosciences) and quantified with ImageStudio6.0. The % GST pulldown was calculated by comparing signal intensities of p140Cap or tau in the input (10 μg) with the corresponding pull-down signal intensity from 0.5 mg protein and then multiplying by 100.

### Co-IP assay

N2a-p140Cap stable cells were plated at a density of 1 × 10^6^ cells in 60 mm dishes. Twenty-four hours after plating, cells were transfected with plasmids encoding Cyto TID or BIN1 TID fusion protein (56) using Lipofectamine 3000 (Invitrogen) and incubated for 24 h before harvesting. The cells were washed twice in ice-cold 1X DPBS and lysed in cold co-IP buffer ([20 mM Hepes-KOH, pH 7.8, 50 mM KCl, 150 mM NaCl, 1 mM EDTA, 1% IGEPAL]) containing freshly added protease inhibitor cocktail (Thermo Scientific) and 0.25 mM PMSF. Clarified lysates were incubated with 3 μl mouse anti-V5 mAb (Thermo Fisher Scientific #R96025) on a nutator at 4 °C overnight. Immune complexes were captured on protein G agarose, which was washed twice in a buffer containing 20 mM Hepes-KOH, pH 7.8, 50 mM KCl, 150 mM NaCl, 1 mM EDTA, and 1.5% IGEPAL. The agarose beads were resuspended in Laemmli buffer and incubated at 95 °C for 5 min before fractionating through 4 to 20% SDS-PAGE gels and analyzed by immunoblotting. A 1/20th aliquot of the original lysate was loaded onto the gel to evaluate the efficiency of co-IP. Membranes were probed with antibodies against BIN1 (Protein Tech #14647-1-AP; 1:1000; validated using BIN1-cKO mice) (14) and p140Cap (Defilippi’s lab mAb; 1:2000) (44).

## Data availability

All the data associated with this study are available in the article or the supplementary material.

## Supporting information

This article contains supporting information.

## Conflict of interest

The authors declare that they have no conflicts of interest with the contents of this article.
