# Supplementary material for: A biophysical and molecular characterization of the interaction between the Alzheimer risk factor BIN1 and the neuronal scaffold protein p140Cap
Source: J Biol Chem. 2025 Aug 31;301(10):110665. doi: 10.1016/j.jbc.2025.110665 (PMC12510028; doi:10.1016/j.jbc.2025.110665)
Supplement: Supporting Figure S1 [file mmc1.pdf]

Coomassie Blue SDS-PAGE

A

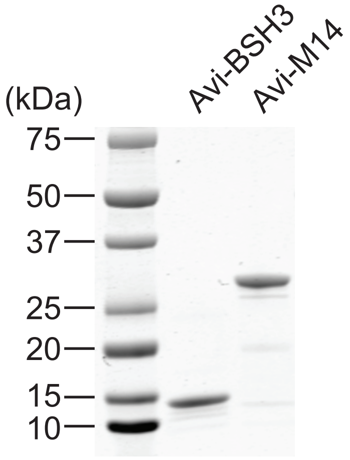

	Contaminate	Protein	Total Area	Purity
Avi-BSH3	0.00483	3.42	3.43	99.7%
Avi-M14	0.00187	0.0971	0.0989	98.2%

Fig. S1. Purified ligands immobilized in SPR experiments. Coomassie blue-stained gel of the biotinylated experimental BIN1 SH3 domain and the control M14 protein, both purified as ligands for SPR experiments. Purity was assessed using ImageJ software to quantify the intensity of the target protein band relative to all contaminants. Each protein was purified to >95%.
