# Supplementary material for: A biophysical and molecular characterization of the interaction between the Alzheimer risk factor BIN1 and the neuronal scaffold protein p140Cap
Source: J Biol Chem. 2025 Aug 31;301(10):110665. doi: 10.1016/j.jbc.2025.110665 (PMC12510028; doi:10.1016/j.jbc.2025.110665)
Supplement: Supporting Figure S2 [file mmc2.pdf]

GST BIN1-SH3 Domain Mutant Construct Sequences

A

	1	11	21	31	41
**WT SH3**	$_{513}$GRLDLPPGFM	FKVQAQHDYT	AT**D**T**DE**LQL**K**	AGDVVLVIPF	QNP**EE**P**DE**GW$_{562}$
**MutA**	$_{513}$GRLDLPPGFM	FKVQAQHDYT	AT**N**T**NQ**LQLK	AGDVVLVIPF	QNPEEPDEGW$_{562}$
**MutB**	$_{513}$GRLDLPPGFM	FKVQAQHDYT	ATDTDELQLK	AGDVVLVIPF	QNP**QQ**P**NQ**GW$_{562}$
**MutK**	$_{513}$GRLDLPPGFM	FKVQAQHDYT	ATDTDELQL**R**	AGDVVLVIPF	QNPEEPDEGW$_{562}$

Fig. S2. Sequences of the wild-type and mutant BIN1-SH3 domain constructs generated as GST fusion proteins. A, The amino acid sequence corresponding to the N-terminal region of the SH3 domain (residues 513-562) from the human BIN1 isoform 1 (NP_647593) is displayed, where Gly at position 1 indicates the first residue of the SH3 domain. Acidic clusters corresponding to MutA and MutB are illustrated in blue, and a basic lysine residue corresponding to the rare GWAS BIN1 coding SNP (rs138047593) is depicted in red. The sequences of the MutA, MutB, and MutK constructs are displayed below the WT sequence with the mutated residues bolded and underlined. The entire SH3 domain (residues 513-593) of each construct was purified for GST pull-down experiments.
