# Supplementary material for: A biophysical and molecular characterization of the interaction between the Alzheimer risk factor BIN1 and the neuronal scaffold protein p140Cap
Source: J Biol Chem. 2025 Aug 31;301(10):110665. doi: 10.1016/j.jbc.2025.110665 (PMC12510028; doi:10.1016/j.jbc.2025.110665)
Supplement: Supporting Figure S3 [file mmc3.pdf]

A

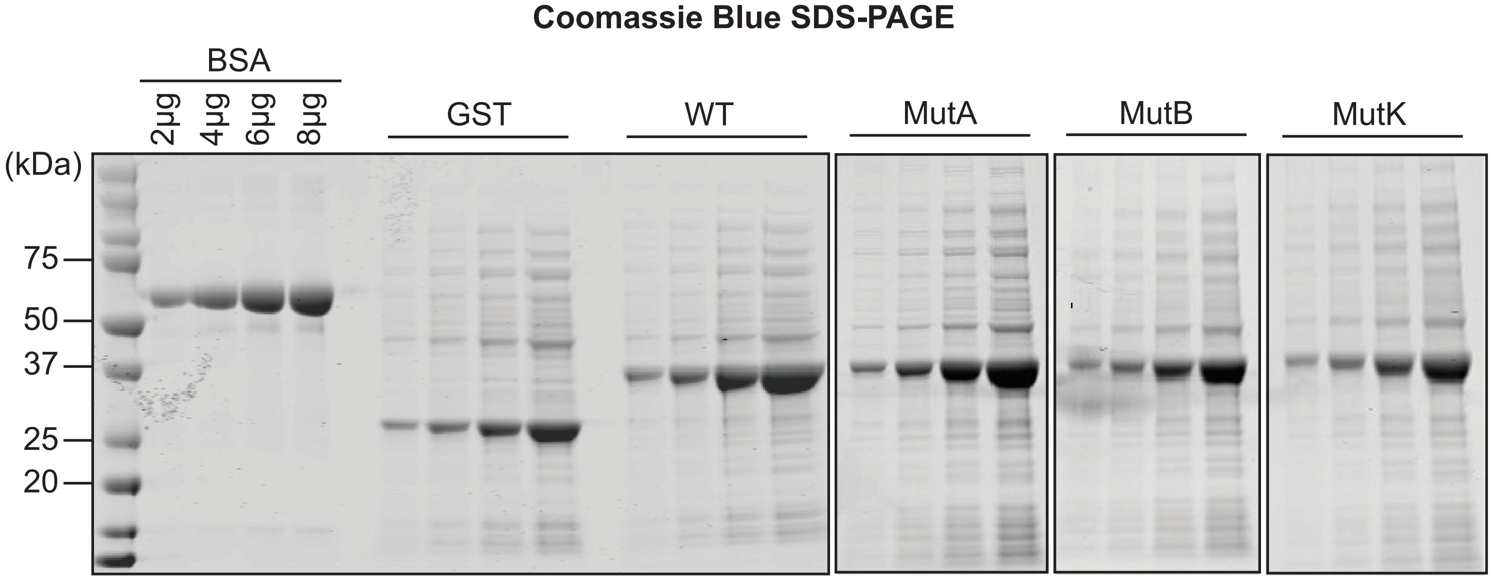

Fig. S3. Purification of GST fusion proteins used for pull-down experiments. Aliquots (5, 10, 20, and 40 μl) of purified GST, GST-SH3 WT, and GST-SH3 experimental mutant protein preparations were fractionated on SDS gels and stained with Coomassie blue. Known amounts (2, 4, 6, and 8 μg) of BSA were loaded onto each gel (not shown) to aid in quantifying GST protein concentrations.
