# Supplementary material for: A biophysical and molecular characterization of the interaction between the Alzheimer risk factor BIN1 and the neuronal scaffold protein p140Cap
Source: J Biol Chem. 2025 Aug 31;301(10):110665. doi: 10.1016/j.jbc.2025.110665 (PMC12510028; doi:10.1016/j.jbc.2025.110665)
Supplement: Supporting Figure S4 [file mmc4.pdf]

N2a-p140Cap Stable Cell Generation

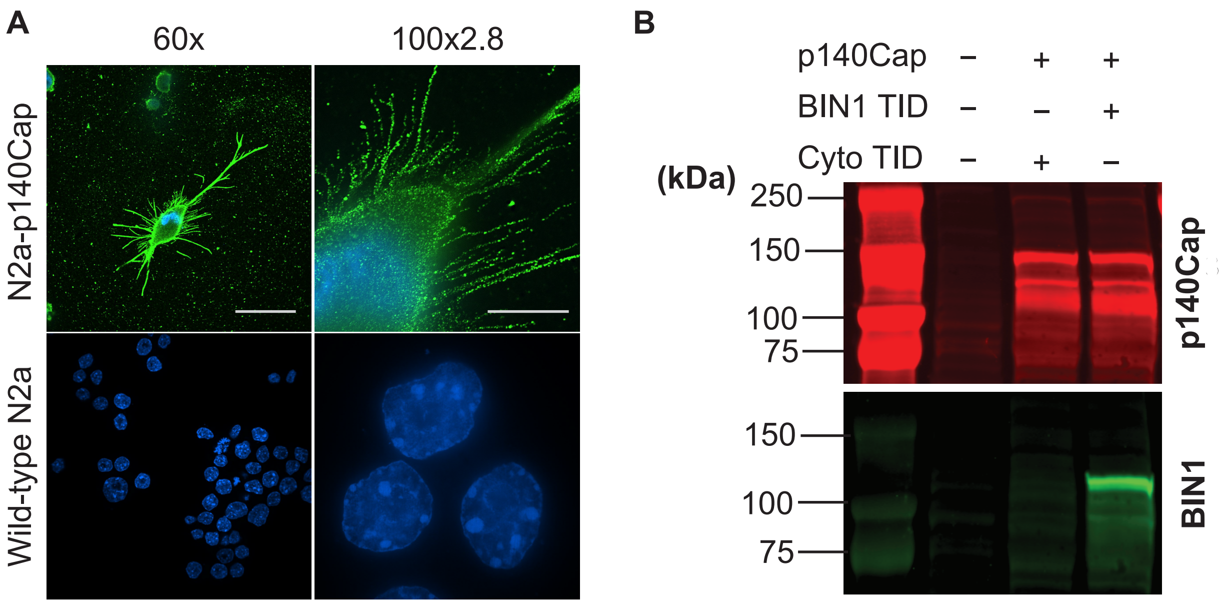

Fig. S4. Characterization of N2a-p140Cap stable cells. A, parental N2a cells and stable pools expressing p140Cap-myc, grown on coverslips, were fixed and stained with mAb 9E10. Images were captured using a 60x objective or a 100x objective with 2.8 SoRa magnification images (scale bars are 50 µm and 10 µm, respectively). B, Western blot analysis of parental N2a, N2a-p140Cap cells transfected with CytoTID, and N2a-p140Cap cells transfected with BIN1TID. The membrane was probed using BIN1 (green) and 9E10 antibodies (p140Cap; red).
