# Supplementary material for: A biophysical and molecular characterization of the interaction between the Alzheimer risk factor BIN1 and the neuronal scaffold protein p140Cap
Source: J Biol Chem. 2025 Aug 31;301(10):110665. doi: 10.1016/j.jbc.2025.110665 (PMC12510028; doi:10.1016/j.jbc.2025.110665)
Supplement: Supporting Figure S5 [file mmc5.pdf]

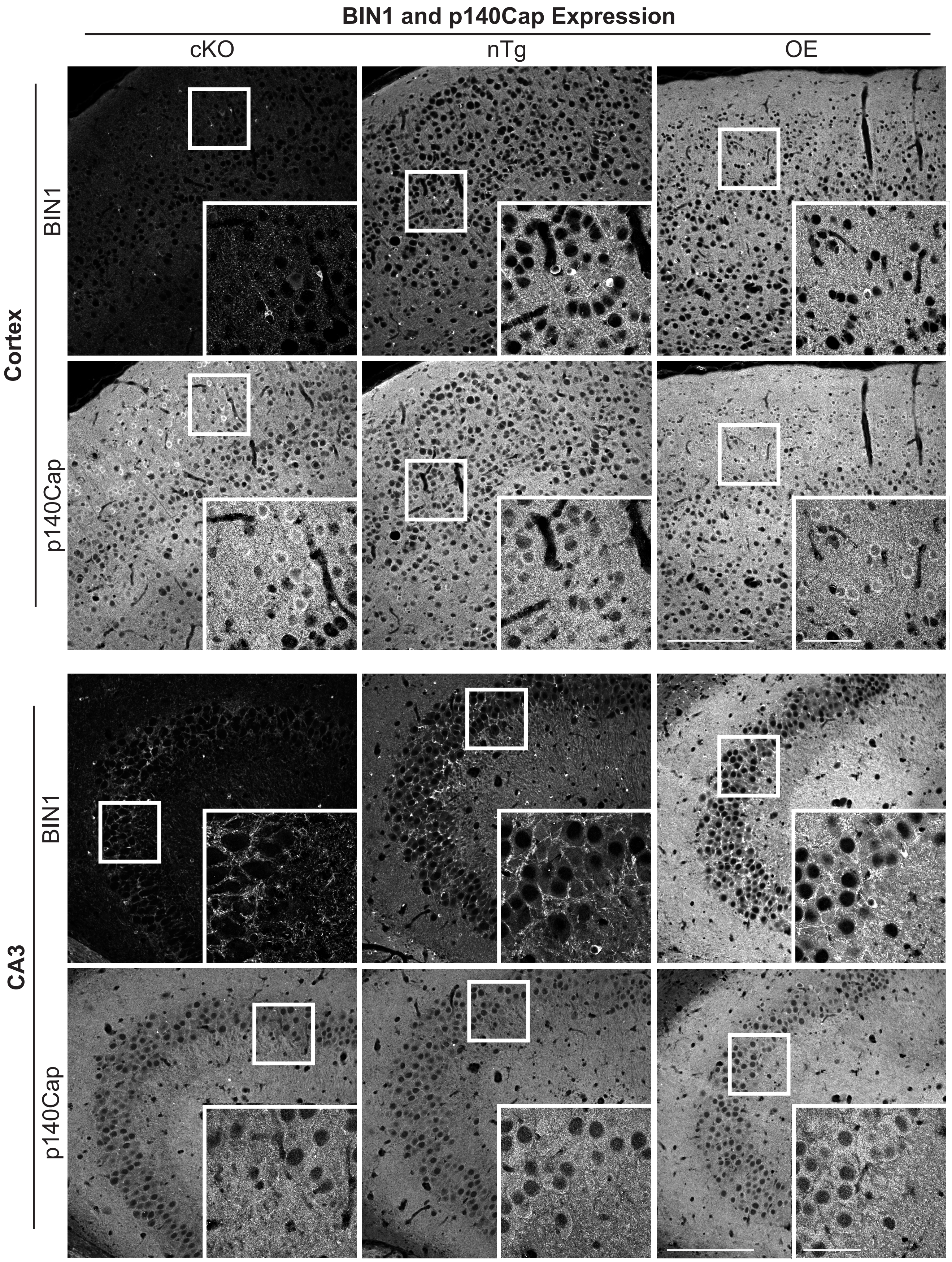

Fig. S5. Bin1 and p140Cap expression in the brains of mice expressing varying levels of BIN1. Representative grey-scale immunofluorescence images from the cortex and CA3 of BIN1-cKO, nTg, and BIN1-OE mouse brains immunostained with antibodies against pAb BIN1 and mAb p140Cap. Images were acquired using a 20X objective (scale bar = 200 µm). The boxed regions are shown as a higher magnification image, acquired using a 100x objective (scale bar = 50 µm).
